# Nanomaterial-Enabled Enhancements in Thylakoid-Based Biofuel Cells

**DOI:** 10.3390/nano15141092

**Published:** 2025-07-14

**Authors:** Amit Sarode, Gymama Slaughter

**Affiliations:** 1 Center for Bioelectronics, Old Dominion University, Norfolk, VA 23508, USA; asaro001@odu.edu; 2 Department of Electrical and Computer Engineering, Old Dominion University, Norfolk, VA 23529, USA

**Keywords:** thylakoid membranes, biofuel cells, graphene-derived nanomaterials, metal oxide, conductive polymers, solar energy harvesting

## Abstract

Thylakoid-based photosynthetic biofuel cells (TBFCs) harness the inherent light-driven electron transfer pathways of photosynthesis to enable sustainable solar-to-electrical energy conversion. While TBFCs offer a unique route toward biohybrid energy systems, their practical deployment is hindered by sluggish electron transfer kinetics, unstable redox mediators, and inefficient interfacing between biological and electrode components. This review critically examines recent advances in TBFCs, with a focus on three key surface engineering strategies: (i) incorporation of nanostructured materials to enhance electrode conductivity and surface area; (ii) application of redox mediators to facilitate charge transfer between photosynthetic proteins and electrodes; and (iii) functional exploitation of individual thylakoid components, including Photosystem I (PSI) and Photosystem II (PSII), to augment photogenerated current output. By systematically evaluating current advancements, this review highlights the synergistic role of materials and biological components in advancing TBFC technology and offers insights into next generation biohybrid solar energy systems with enhanced efficiency and scalability.

## 1. Introduction

The accelerating global energy demand, coupled with the increasing threat of climate change, has intensified efforts to develop renewable energy technologies that are efficient, scalable, and environmentally benign. Solar energy, by virtue of its ubiquity and sustainability, stands at the forefront of these efforts. However, while conventional photovoltaic technologies, primarily based on silicon semiconductors, have seen great progress, their fabrication costs, energy payback time, and relatively modest efficiencies under diffuse light remain persistent drawbacks [1]. In contrast, photosynthesis, nature’s blueprint for solar energy capture, exhibits quantum efficiencies approaching unity during the initial stages of light harvesting and charge separation [2]. This exceptional efficiency, achieved through evolutionarily optimized protein–pigment complexes, has inspired the development of photosynthesis-based energy conversion systems that aim to merge biological and synthetic elements for sustainable electricity generation.

Photosynthesis-based biofuel cells (PBFCs) are emerging as a promising class of photo-bioelectrochemical devices that exploit the photochemical energy transduction of photosynthetic organisms or their isolated components, mostly thylakoid membranes, Photosystem I (PSI), and Photosystem II (PSII). To contextualize emerging design strategies, we propose a classification framework for thylakoid-based photosynthetic biofuel cells (TBFCs). Full TBFCs employ intact thylakoid membranes containing the complete native photosynthetic electron transport chain, PSII, cytochrome b6f, PSI, and associated cofactors embedded within the lipid bilayer. These systems enable multi-site electron extraction but are often constrained by photoinstability, membrane orientation, and diffusion-limited charge transport. Subcomponent systems, including PSII-only and PSI-only configurations, isolate individual photosystems to target specific reactions: PSII for light-driven water oxidation and O_2_ evolution, and PSI for high-potential electron transfer to external acceptors. While these approaches enhance redox alignment and charge separation, they sacrifice the self-contained functionality of native thylakoid assemblies. Hybrid systems integrate PSI or PSII with synthetic components such as redox-active polymers, semiconductors, or enzymes to emulate or augment natural charge separation and facilitate direct or mediated electron transfer. This classification provides a unifying framework to evaluate performance trade-offs, mechanistic constraints, and the scalability of diverse TBFC architectures discussed throughout this review.

**Figure 1:**
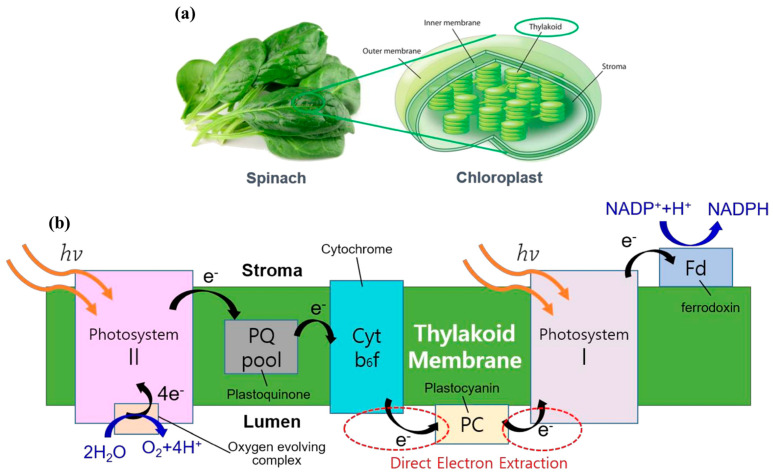
Schematic representation of (**a**) chloroplasts showing thylakoid membranes as sites of light-dependent reactions. (**b**) Photosynthetic electron transport chain within the thylakoid membrane, highlighting key redox components and potential sites for electron extraction in bioelectronic applications [3].

Among these, TBFCs are of particular interest due to the structural and functional completeness of thylakoid membranes. Embedded within a lipid bilayer, thylakoids harbor the full complement of protein complexes required for light-driven electron transport, including PSII, cytochrome b6f, PSI, plastoquinone (PQ), plastocyanin (PC), ferredoxin (Fd), and associated electron shuttles. This intact system offers a unique platform for solar-to-electrical energy conversion, where photogenerated electrons can be extracted at multiple points along the electron transport chain, as illustrated in Figure 1 [3]. Figure 1a depicts isolated chloroplasts from spinach leaves containing thylakoid stacks, while Figure 1b details the linear electron transport chain within the thylakoid membrane. Upon light absorption (hv), PSII catalyzes the oxidation of water to oxygen, releasing protons and electrons that traverse the PQ pool, cytochrome b6f complex, and PC before reaching PSI. A second photon excites PSI, resulting in high-energy electrons that reduce NADP^+^ to NADPH via Fd and ferredoxin-NADP^+^ reductase. The red dashed circles mark key nodes where electrons can be intercepted for bioelectronic applications. These electron harvesting sites are critical to TBFC design, enabling semi-artificial systems that convert solar energy into usable electrical power.

Recent efforts have focused on improving the performance and stability of TBFCs by addressing core challenges related to charge extraction, material–biological interface engineering, and long-term operational robustness. Strategies such as nanostructuring electrode surfaces, incorporating redox mediators, and optimizing substrate architectures have yielded significant performance gains. For example, TBFCs employing carbon nanotube (CNT)-modified electrodes have demonstrated photocurrent densities up to 68 µA/cm^2^ under visible light [4], while biophotovoltaic systems with live photosynthetic organisms (e.g., *Synechocystis* sp. PCC6803, Chlorella vulgaris) have achieved peak power densities exceeding 1900 mW/m^2^ [5]. Despite these advances, major barriers remain, including inefficient direct electron transfer (DET), redox mediator instability, electrode biofouling, and degradation of photosynthetic activity over time.

While previous reviews have broadly surveyed biohybrid PBFC systems or focused primarily on PSI- and PSII-based platforms, this review uniquely centers on TBFCs and critically evaluating recent progress and identifying four key surface engineering strategies that have demonstrated the greatest potential to overcome longstanding bottlenecks in TBFC development. First, we examine how nanomaterials and advanced electrode modifications enhance bioelectronic communication, structural stability, and current density by improving surface-to-volume ratios and enabling more effective integration of thylakoid membranes with conductive scaffolds. Second, we assess the selection, tuning, and limitations of redox mediators in facilitating efficient charge transfer across the bio–abiotic interface. Finally, we investigate efforts to decouple and reconstitute individual components of the thylakoid membrane, particularly PSI and PSII, as modular catalytic units with tunable photochemical and electrochemical properties. In integrating these three areas, this review provides a novel and in-depth perspective on the synergy between materials and photosynthetic bioengineering in advancing TBFC technology. Our discussion highlights the importance of electrode architecture, electron transfer dynamics, and biological interface design in achieving high-efficiency solar-to-electrical conversion. Moreover, we identify persistent limitations such as operational stability, mediator toxicity, and scale-up feasibility, and outline future research directions that may lead to robust, durable, and commercially viable TBFC platforms.

### Literature Selection Methodology

This review is based on a structured literature search conducted across Scopus, Web of Science, and Google Scholar, spanning January 2010 to April 2025. Search terms included combinations such as “thylakoid biofuel cells”, “photosynthetic bioelectrochemical cells”, “PSI/PSII electron transfer”, “direct electron transfer”, and “redox mediators in PBFCs”. Selection was limited to peer-reviewed original articles and critical reviews directly addressing TBFC systems or key components such as redox interfaces, nanomaterial scaffolds, or photosystem–electrode coupling. Priority was given to studies reporting quantitative performance metrics (e.g., photocurrent density, power output) and experimental insight into materials design and charge transfer mechanisms. Excluded were non-peer-reviewed sources and studies lacking relevance to photosynthetic systems or TBFC performance optimization. This approach ensures that the included literature reflects both foundational advances and emerging strategies central to TBFC development.

## 2. Nanostructured Interfaces for Enhanced Performance in Thylakoid Biofuel Cells (TBFCs)

Nanomaterials have emerged as indispensable components in advancing TBFCs, offering innovative solutions to long-standing challenges such as inefficient electron transfer, poor biointerface stability, and suboptimal energy conversion efficiency. The incorporation of functional nanostructures particularly carbon-based materials, metal oxides, and conductive polymers has improved thylakoid immobilization, enhanced charge transport pathways, and increased both photocurrent and power output. These performance gains are intrinsically linked to the physicochemical properties of the nanomaterials, including particle size, surface area, porosity, crystallinity, and nanoscale architecture. Such characteristics not only determine the extent of thylakoid–electrode interaction but also govern redox alignment, electron mobility, and operational durability.

### 2.1. Carbon-Based Electrode Interfaces for Thylakoid Integration

Carbon-based materials have emerged as an excellent platform for TBFC electrodes due to their chemical inertness, biocompatibility, conductivity, and structural tunability. Lettieri et al. illustrated the feasibility of using untreated carbon paper as a support for pea-derived thylakoid membranes in a mediator-free system [6]. This minimalist design preserved the intrinsic electron transfer activity of the thylakoid membranes and achieved photocurrent densities of up to 14 μA/cm^2^. Additionally, the system demonstrated herbicide sensing capabilities by detecting PSII inhibitors such as diuron, terbuthylazine, and metribuzin at submicromolar levels, with notable signal retention over two weeks of cold storage. Despite their limited ability to DET in the absence of further material modifications, carbon substrates are suitable for low-cost, passive interfacing with thylakoid membranes in solar-energy harvesting and environmental monitoring applications.

**Figure 2:**
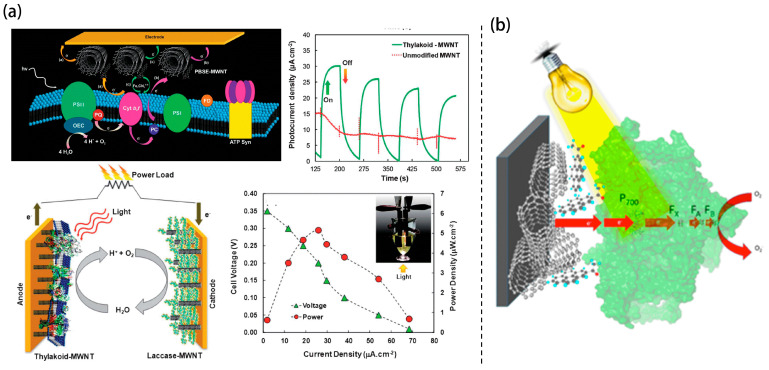
Schematic representation of (**a**) TBFCs with MWCNT-enhanced architecture [4]. (**b**) Photo-induced electron transfer in a PSI-based cathode, where PSI is immobilized on a MWCNT-modified electrode via a pyrene linker to facilitate light-driven charge transport [13].

Transitioning from planar to high-surface-area scaffolds, three-dimensional (3D) carbon nanostructures have gained prominence due to their superior electrochemical surface area, improved charge transport pathways, and capacity for dense biocatalyst loading. Calkins et al. demonstrated early success in functionalizing multi-walled carbon nanotubes (MWCNTs) with thylakoid membranes via molecular tethering Figure 2a [4]. The high aspect ratio and conductive network of the MWCNTs facilitated DET from PSII-driven water oxidation in a complete cell using this bioanode and a laccase cathode, which yielded a photocurrent density of 68 µA/cm^2^ and peak power output of 5.3 µW/cm^2^ under illumination. These photocurrent density exceeded earlier photosynthetic systems [7,8,9,10,11].

Thereby, these nanocarbon scaffolds with controlled dimensions and defect densities can create highly effective electron transfer pathways while maintaining thylakoid activity. In a related effort, Yun et al. emphasize the role of internal electrical connectivity and surface functionalization in enhancing bioanode architecture by employing mussel adhesive protein-coated CNTs (MAP-CNTs) [12]. This approach improved CNT dispersion and established strong electrostatic interactions with negatively charged thylakoid membranes. The resulting conductive percolation network enabled mediator-free photocurrent generation of 4.25 μA/cm^2^, an order of magnitude improvement over traditional systems.

Expanding on this concept, Christwardana et al. embedded chlorophyll extracted from *Chlorella vulgaris* into CNT frameworks [14]. The hydrophobic tail of chlorophyll facilitated intimate interactions with the CNT surface, enabling efficient photoinduced electron transfer. The chlorophyll-CNT photoanode reached a photocurrent of 10.22 mA/m^2^ and a power density of 9.48 mW/m^2^, emphasizing the synergistic effect of natural photosensitizers and conductive nanostructures in low-cost, high-performance biohybrid systems. Further studies by Pankratov et al. revealed that the surface chemistry of CNTs, specifically the carbon-to-oxygen (C/O) ratio, affects TBFC performance [15]. Higher C/O ratios led to increased photocurrents (97.1 ± 8.3 μA cm^−2^) under illumination. However, this configuration had reduced long-term stability, indicating a trade-off between performance and durability based on nanomaterial surface oxidation states.

Efforts in the domain of flexible and printable electronics have enabled the realization of scalable, lightweight, and disposable TBFC platforms. Son et al. leveraged inkjet printing techniques to deposit CNT electrodes and thylakoid membranes directly onto porous paper substrates [16]. The CNT films exhibited excellent conductivity (117.3 Ω/sq), while the inherent porosity of paper promoted enhanced electrolyte penetration and mass transport. Under simulated solar irradiation, the device produced a photocurrent of 4.8 mA/m^2^ and a peak power density of 250 μW/m^2^ surpassing algae-based platforms by over 20-fold. This approach demonstrated that printability and mechanical flexibility need not come at the expense of electrochemical performance, especially when materials are carefully selected to complement thylakoid membrane architecture and reaction kinetics.

Beyond photo-bioanodes, Ciornii et al. developed a PSI-based photo-biocathode by immobilizing cyanobacterial PSI onto MWCNT-modified glassy carbon electrodes using pyrene linkers as depicted in Figure 2b [13]. The quasi-2D nature of the nanotubes enhanced electronic coupling, and cytochrome c further facilitated electron mediation. This design yielded cathodic photocurrents up to 18 μA/cm^2^, revealing how precise nanoscale organization and anchoring chemistry directly modulate charge injection kinetics and device performance.

### 2.2. Reduced Graphene Oxide and Mxene for Enhancing Thylakoid–Electrode Coupling

Building on the demonstrated feasibility of interfacing thylakoid membranes with conductive substrates, subsequent efforts have focused on developing scalable 3D architectures to further enhance thylakoid–electrode interactions and overall device performance. Morlock et al. advanced this concept through a scalable 3D photo-bioanode architecture composed of reduced graphene oxide (rGO) framework fabricated via a vacuum filtration technique [17]. The porous and flexible rGO scaffold enabled close contact between thylakoid membranes and the conductive matrix, leading to improved light penetration, high electroactive surface area, and photocurrent densities up to 50 μA cm^−2^. The hierarchical porosity and controlled surface roughness facilitated both mass transport and electron flow in TBFCs. In another study, Pankratova et al. fabricated a 3D electrode using electrochemically rGO followed by aminoaryl functionalization, enabling a biomimetic interface that facilitated DET from spinach-derived thylakoid membranes [18]. The resulting electrode delivered a photocurrent density of 5.24 ± 0.50 μA/cm^2^ among the highest reported under mediator-free conditions and, when paired with an enzymatic oxygen-reducing cathode, generated a power output of 1.79 ± 0.19 μW/cm^2^. Mechanistically, the functionalized graphene architecture improved charge delocalization and facilitated strong electrostatic interactions with the negatively charged thylakoid membranes, enhancing their orientation and electronic coupling to the electrode surface. This nanostructuring of the bioanode surface bridges the performance gap between mediated electron transfer (MET) and DET configurations by providing intimate contact and optimized redox alignment.

**Figure 3:**
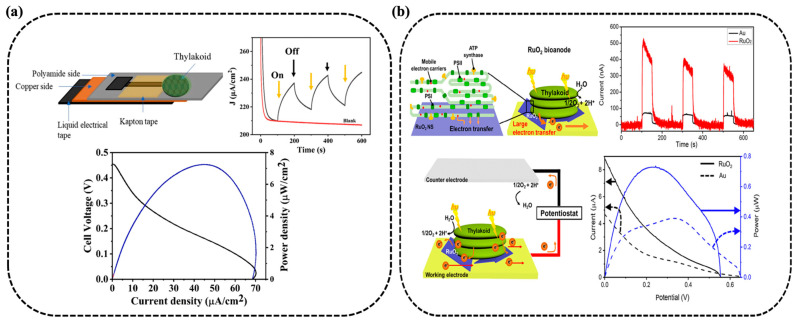
TBFCs with enhanced architectures. (**a**,**b**) Configurations using flexible LIG-MXene substrates [19] and RuO_2_ nanosheets [22].

Recent work has turned toward enhancing performance through advanced material integration strategies. As illustrated in Figure 3a, Sarode et al. explored 2D material integration by combining thylakoid membranes with Nb_4_C_3_T_x_ MXene-decorated laser-induced graphene (LIG) [19]. The porous, high-surface-area scaffold promoted efficient thylakoid membrane immobilization and robust charge transfer. This light-harvesting bioanode paired with a gas diffusion platinum cathode generated an open circuit voltage of 0.45 V, a photocurrent of 68.69 μA/cm^2^ and power density of 7.24 μW/cm^2^. The uniformity and conductivity of MXene, alongside the tailored porosity of LIG, demonstrate the importance of engineering nano–bio interfaces with tunable microstructures for maximum energy conversion.

### 2.3. Semiconductor and Hybrid Nanostructures for Enhancing Light Harvesting and Charge Transfer

To overcome the limitations of carbon-based electrodes, photoactive semiconductor materials that can synergistically enhance light harvesting while facilitating charge separation have been explored for solar energy capture with biological catalysis. Amao et al. designed a biohybrid platform by immobilizing *Spirulina platensis* thylakoid membranes onto nanocrystalline titanium dioxide (TiO_2_) electrodes [20]. The photoactive TiO_2_ served as a light-absorbing scaffold that enabled the dual function of oxygen evolution and formate generation in a CO_2_-saturated environment. When paired with a viologen-mediated cathode housing formate dehydrogenase, this system achieved a short-circuit photocurrent of approximately 50 μA/cm^2^, while stoichiometric analyses confirmed efficient redox cycling. This design merged the photonic and biochemical domains, indicating that semiconductor–thylakoid membrane interfaces hold promise for solar-to-chemical conversion and carbon valorization strategies. Additional design strategies incorporate conductive polymers such as polyaniline (PAni) with PSI onto TiO_2_ thin films bioanode [21]. The electropolymerized PAni matrix offers a 3D conductive network that enhances PSI loading and facilitates charge transfer. This design improves both the photocurrent response and operational stability under ambient conditions.

**Figure 4:**
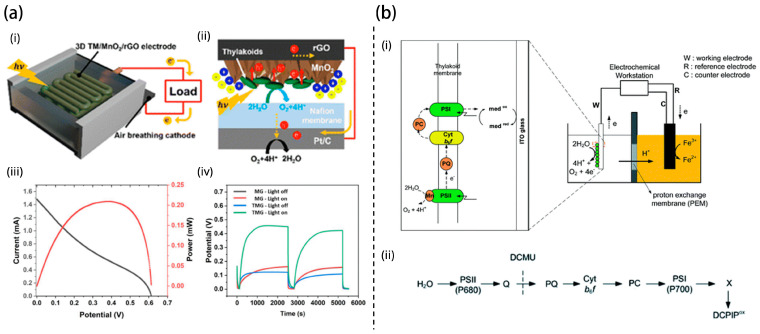
Schematic of (**a**) biophotovoltaic cell, (**i**) 3D-printed thylakoid membrane/MnO_2_/rGO photoanode and air-breathing Pt/C cathode. (**ii**) Electron extraction from thylakoid membranes on MnO_2_/rGO. (**iii**) I–V and power density curve. (**iv**) Light-triggered OCV profiles demonstrating self-charging in TMG electrodes [23]. (**b**) Two-chamber photosynthetic electrochemical cell, (**i**) Thylakoid-based photoelectrochemical cell setup. (**ii**) Native electron transport chain illustrating light-driven electron flow, mediator interaction, and 3-(3,4-dichlorophenyl)-1,1-dimethylurea (DCMU) inhibition site [25].

Ruthenium dioxide (RuO_2_) nanosheets were employed by Hong et al. to enhance thylakoid adhesion and redox molecule docking through proton-adsorbing and polar surfaces (Figure 3b) [22]. The resulting bioanode produced a fivefold increase in photocurrent and a fourfold increase in power density compared to conventional gold-based systems. The crystalline nature and high specific surface energy of the RuO_2_ nanosheets likely contributed to enhanced catalytic activity and redox interactions, bridging the bioinorganic interface. This system was further demonstrated to power small electronic devices, demonstrating the feasibility of TBFCs for low-power applications. In a separate approach, Jung et al. developed MnO_2_-decorated, porous 3D-printed graphene electrodes that enhanced thylakoid adhesion and electron extraction [23]. Optimized MnO_2_ deposition tuning porosity and crystallinity led to photosynthetic electron (PE) current densities of 38.2 μA/cm^2^, with peak outputs surpassing 100 μA/cm^2^ under visible light illumination (Figure 4a). This reinforces the value of integrating redox-active metal oxides with architected carbon frameworks for photoresponsive energy storage and harvesting.

In addition, Lee et al. reported one of the highest photocurrents in TBFCs to date by assembling thylakoid membranes with indium tin oxide nanoparticles (ITOnp) and osmium-based redox polymers (Os-RP) on porous graphite [24]. The precise material ratio (TM/Os-RP/ITOnp = 1:0.5:30) was critical, yielding 0.5 mA/cm^2^ photocurrent and 122 μW/cm^2^ power output. Mechanistic studies confirmed that PSII was the primary source of electrons in this system, further illustrating how nanoscale composition and stoichiometry influence electron extraction and redox compatibility at the interface.

Since nanoscale composition and stoichiometry have been shown to influence electron transfer, subsequent research has focused on how electrode morphology and configuration impact the efficiency of thylakoid-based photoelectrochemical systems. Dewi et al. explored how electrode morphology affects electron transfer from thylakoid membranes using ITO in a two-chamber photosynthetic electrochemical cell (PEC) shown in Figure 4b [25]. This study effectively demonstrates the feasibility of integrating thylakoid membranes with ITO electrodes for biohybrid photoelectrochemical devices by leveraging photosynthetic water splitting. Photocurrent measurements revealed that thylakoids physically adsorbed on ITO produced significantly higher output than dispersed configurations, emphasizing the importance of membrane–electrode proximity. Spectral data confirmed the retention of functional light-harvesting complexes post-deposition, while inhibitor and mediator experiments elucidated PSI’s role in electron transfer. The observation of bi-directional photocurrents under varying potentials highlights the dynamic charge transport behavior of the system. While the findings validate a promising platform for sustainable energy conversion, long-term stability, and efficiency optimization remain critical challenges for practical deployment.

### 2.4. Molecular and Multidimensional Interface Engineering for Enhanced Charge Transfer in TBFCs

An innovative approach to achieving efficient DET was demonstrated by Hamidi et al., who employed osmium-based redox polymers to create a conductive interface between intact thylakoid membranes and graphite electrodes [26]. This strategy circumvented the need for isolated reaction centers, preserving the native membrane structure while facilitating electron transfer via redox polymers with tailored formal potentials. Of the various polymers tested, osmium(II) bis(2,2′-bipyridine) polyvinylimidazole chloride complex [Os(bpy)_2_(PVI)_10_Cl]^+/2+^, (Os-4) exhibited the highest redox potential (0.441 V vs. standard hydrogen electrode (SHE) and enabled a photocurrent density of 42.4 μA/cm^2^. Inhibition experiments using diuron confirmed that the electron flow predominantly originated from PSII, validating the effectiveness of the redox polymer interface in extracting photogenerated electrons from functional photosynthetic sites. The high redox potential of Os-4 enabled favorable alignment with PSII’s plastoquinone binding site, thereby facilitating unidirectional electron transfer and minimizing recombination events. In addition, the strong coordination between the polymer matrix and thylakoid membrane proteins also conferred structural stability and prolonged activity, highlighting the dual role of redox polymers as both electronic mediators and stabilizing agents.

Expanding beyond structural and compositional tuning of electrode materials, molecular-level strategies to modulate interfacial interactions and enhance charge transfer efficiency in thylakoid-based systems are being explored. Kirchhofer et al. introduced a novel molecular design strategy by conjugating oligoelectrolytes (COEs) with varied length and charge into thylakoid membranes [27]. These non-redox-active molecules modulated interfacial interactions and increased photocurrent by 2.3-fold without altering the photosynthetic function. The ability to modulate electronic coupling at the molecular level presents a promising avenue for interfacial optimization without compromising the photosynthetic machinery. And thus, it results in the improved electron transfer across the bioelectrode interface.

Sarode and Slaughter introduced a multidimensional electrode design that not only enhances charge transfer but also demonstrates practical energy harvesting capabilities. Sarode et al. present an advancement in biohybrid photoelectrochemical fuel cells by integrating LIG, PEDOT, and isolated thylakoid membranes into a hierarchically structured photo-bioanode, demonstrating both fundamental innovation and practical viability [28]. The LIG’s high surface area and electron mobility synergistically coupled with PEDOT’s redox activity and biointerface compatibility created an efficient platform for thylakoid immobilization and light-driven electron transfer. This dual-conductive architecture facilitates robust charge separation and rapid transport, resulting in a significant photocurrent density (18 µA/cm^2^) and peak power output (36 µW/cm^2^), surpassing conventional systems reliant on more complex or costly nanomaterials like MXenes. Beyond performance metrics, the practical applicability of the system was demonstrated by powering a low-voltage LED through an integrated charge pump circuit, marking an important step toward autonomous, self-powered bioelectronic devices. However, while the pulsed LED illumination illustrates potential utility, the transient nature of current output remains a limiting factor for continuous operation. Future work could benefit from exploring strategies to stabilize and extend charge delivery, potentially by integrating energy storage elements. Overall, the study offers a robust proof-of-concept and sets a benchmark for the scalable, sustainable design of bioelectrochemical energy platforms.

The use of nanomaterials in the design and development of TBFCs continues to garner significant attention. A clear correlation emerges between the physicochemical properties of nanomaterials and the enhancement of TBFC performance metrics. Specifically, surface area and morphology play a central role in enhancing thylakoid membrane immobilization and maximizing biocatalytic contact while the redox alignment between the electrode and thylakoid redox centers, either via polymer engineering or material selection, critically determines the efficiency of electron harvesting. Although conductive polymers (e.g., PEDOT, PAni) offer unique flexibility and biocompatibility advantages for printable and wearable TBFCs, they typically produce moderate photocurrents (15–40 μA/cm^2^), but their low cost, tunable redox properties, and excellent integration with biological films make them ideal for disposable or flexible devices [29]. Moreover, the mechanical and environmental stability can be achieved through covalent or non-covalent immobilization strategies and synergistic interactions with redox mediators or photosystems that preserve bioactivity while supporting repeatable operation. The move toward flexible, printable, and disposable materials opens the door for integrated, real-world TBFC applications in environmental sensing, point-of-care diagnostics, and low-power energy harvesting.

## 3. Redox Mediators as Critical Interfaces in Thylakoid-Based Biofuel Cells

The central challenge in TBFC design lies in the integration of biological and electrochemical domains: specifically, enabling efficient and sustained extraction of photoexcited electrons generated by photosynthetic components such as PSII, cytochrome b6f, and PSI. While the thylakoid membrane’s internal electron transport chain offers a natural platform for solar energy harvesting, direct interfacing with electrodes remains hindered by kinetic and structural mismatches. As a result, the design of electrode materials to facilitate efficient electron transfer at the bioelectrode interface becomes a critical determinant of device performance impacting not only photocurrent generation but also the overall functionality, scalability, and application scope of TBFCs. Redox mediators play a central role in overcoming this limitation by facilitating charge transfer between the photosynthetic machinery and the electrode surface. The choice and design of mediators, ranging from natural cofactors to synthetic molecules and polymeric interfaces, critically influence the kinetics, directionality, and efficiency of electron flow.

### 3.1. Natural Mediators: Chlorophyll A and Cytochrome C

Chlorophyll a and cytochrome c, intrinsic to native photosynthetic and respiratory chains, offer biocompatible electron shuttles that integrate seamlessly with thylakoid electron transport. Their natural alignment with protein complexes allows efficient coupling of light-driven redox events to electrode-based charge harvesting [30]. Chlorophyll a has been shown to support vectorial electron flow within artificial lipid bilayers, enabling directional electron transfer from reduced cytochrome c to oxidize ferredoxin, mirroring native thylakoid function [31].

Moreover, multistep light-induced electron transfer has been demonstrated in reconstituted membrane systems involving cytochrome c and cytochrome c oxidase, showcasing the potential of chlorophyll-mediated redox cascades [32]. Cytochrome c has also emerged as a multifunctional mediator in hybrid systems, where it serves both as a redox shuttle and charge reservoir, enabling pulsed power delivery with significantly elevated current outputs [33]. Bioconjugates of photoactivated cytochrome c have demonstrated stable room-temperature electron transfer [34], highlighting their promise for ambient-operating, light-driven biosystems. Despite these strengths, the limited redox potential tunability of these natural mediators and their susceptibility to photodegradation under prolonged illumination necessitate complementary strategies for performance stabilization.

### 3.2. Synthetic Mediators as Fast and Reversible Electron Shuttles

Viologen derivatives represent one of the most extensively studied classes of synthetic redox mediators in TBFCs due to their fast, reversible redox kinetics and tunable electrochemical properties. Their small molecular size and low redox potential make them ideal for mediating electron transfer from PSII to the electrode. In systems utilizing *Spirulina platensis* thylakoids, viologen compounds facilitated visible-light-driven CO_2_ reduction to formic acid. This demonstrates their ability to couple photosynthetic activity with electrocatalytic carbon conversion [35]. Methyl viologen has also been effectively applied in hybrid systems for H_2_O_2_ generation using immobilized thylakoids or microalgae, reflecting its high electron affinity and compatibility with biological reductants [35].

Moreover, viologen-based systems incorporating spinach thylakoids have demonstrated enzymatic CO_2_/formate interconversion, highlighting their dual functionality in carbon fixation and electron harvesting applications [36]. Despite their strong performance, concerns remain regarding viologen cytotoxicity, mediator leaching, and long-term redox stability, prompting efforts to anchor or encapsulate these mediators within protective matrices or hydrogels.

### 3.3. Design of Mediators via Redox Potential Engineering

Redox potential alignment between the mediator, thylakoid protein complexes (e.g., PSI/PSII), and the electrode is a key determinant of charge transfer efficiency. Thus, redox mediator design has increasingly incorporated predictive modeling and virtual screening techniques. For example, phenazine and phenothiazine derivatives were computationally screened using machine learning-guided simulations to identify water-soluble, redox-stable candidates with optimized potentials for integration in carbon capture and bioelectrocatalysis platforms [37]. In addition, density functional theory-based simulations have accelerated the identification of phenazine variants with desired redox potentials and stability in aqueous environments [38].

Such computationally guided design frameworks offer a scalable, low-cost route to mediator optimization, eliminating time-consuming trial-and-error experimentation and enabling targeted exploration of novel redox scaffolds.

### 3.4. Polymer-Based Redox Interfaces

Redox-active polymers provide an effective means of immobilizing mediators at the electrode interface while preserving mediator accessibility and reducing diffusion losses. These polymers form electronically conductive matrices that enable localized electron transfer from thylakoids or enzymes to the electrode, thereby improving efficiency and stability. Polypyrrole-based films doped with osmium or ruthenium complexes have enhanced mediator retention and minimized leaching, leading to sustained current generation in laccase-modified biocathodes with power densities reaching 81 µW/cm^2^ and 71 µW/cm^2^, respectively [39]. When applied to TBFCs, osmium-based redox polymer-modified electrodes interfaced with isolated thylakoid membranes exhibited stable photocurrent generation under continuous illumination, reinforcing the value of conductive polymer networks in supporting photoelectrochemical function [39]. These hybrid architectures offer modularity in tailoring redox potential, hydrophilicity, and biocompatibility, all of which are critical parameters for long-term bioelectrode operation.

### 3.5. Redox Regulation of Photosynthetic Enzymes

The activity of thylakoid-associated enzymes, such as carbonic anhydrase (CA), is inherently redox-sensitive and can be modulated by exogenous mediators. In maize thylakoids, ferricyanide suppressed CA activity in dark conditions by oxidizing a pigment component known as “D480”, which regulates bicarbonate binding to PSII. This inhibition was reversed upon illumination, indicating a redox-gated enzymatic mechanism [40]. In Chlamydomonas reinhardtii, a PSII-associated CA (CAH3) plays a vital role in proton removal during water oxidation at low pH, with potential redox regulation through disulfide bridge modulation [41]. Further, redox mediators not only facilitate electron transfer but can also influence the kinetics and stability of auxiliary enzymatic functions integral to photosynthetic energy conversion. Understanding and harnessing these redox-enzyme interactions is essential for optimizing TBFCs under variable light and pH conditions. Overall, the selection and engineering of redox mediators remain central to advancing the performance of TBFCs. Natural mediators offer biocompatibility but limited tunability, while synthetic and polymeric mediators enable fine control over redox potential, stability, and localization.

### 3.6. Redox Mediation Strategies to Overcome Charge Transfer Limitations in TBFCs

In TBFCs, redox mediators are essential for facilitating electron transfer between thylakoid membranes and the electrode interface. While native photosynthetic components can generate photoexcited charge carriers, their direct electronic communication with electrode materials is severely constrained. These limitations stem primarily from the insulating nature of the lipid bilayer, unfavorable spatial orientation of redox-active cofactors, and a lack of robust electrical contact at the bioelectrode interface. Consequently, the inclusion of redox mediators, both soluble and immobilized, has become a critical design strategy to bridge biological charge transport and abiotic energy conversion.

**Figure 5:**
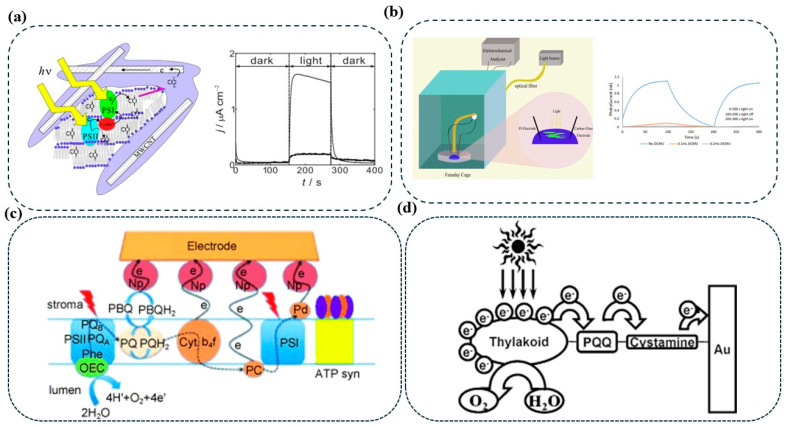
Thylakoid-based photobioanode configurations and electron transfer strategies. (**a**) Photocurrent response and measurement setups under light/dark conditions [42]. (**b**) Experimental setup and measurement setups under light/dark conditions. Electrode wiring via (**c**) PBQ [44] and (**d**) nanoparticles, PQQ, and cystamine to enhance electron transfer from PSI and PSII [45].

To address these constraints, Takeuchi et al. systematically investigated a panel of quinone-based mediators to identify candidates with favorable redox alignment and aerobic stability (Figure 5a) [42]. Using linear free-energy relationship analysis, 1,2-naphthoquinone was identified as a particularly efficient mediator, exhibiting a redox potential that closely matches the terminal electron donors in the thylakoid chain. This compound also displayed enhanced resistance to oxidative degradation. When integrated into a thylakoid membrane/MWCNT-based composite photoanode, 1,2-naphthoquinone facilitated robust electron transfer from spinach thylakoids to the electrode, achieving photocurrent densities exceeding 100 μA/cm^2^ under illumination. This performance improvement is attributed to the mediator’s ability to readily accept electrons from reduced plastoquinone pools or PSI-derived carriers and inject them efficiently into the conductive network, thus bypassing the kinetic bottlenecks of DET.

Quinone-based mediators such as PBQ have also been extensively evaluated for their compatibility with photosynthetic systems. Hasan et al. utilized PBQ in thylakoid membrane–AuNP/Au electrode constructs and achieved photocurrent densities of approximately 130 μA/cm^2^ under visible light (Figure 5b) [43]. PBQ acts as a mimic of natural plastoquinone, accepting electrons from PSII following photoactivation and water oxidation. It is subsequently reduced to hydroquinone (PBQH_2_), which diffuses to the anode where it is reoxidized, completing the redox cycle. This mechanism facilitates efficient electron shuttling between PSII and the electrode, accelerating photocurrent generation and supporting high turnover. Further, the use of gold nanoparticles on the electrode surface enhances local electron transfer kinetics by increasing surface area and facilitating electrochemical coupling. However, PBQ’s utility is constrained by its photoinstability and potential degradation under prolonged illumination, necessitating improved strategies for stabilization and light management.

Similarly, phenazine methosulfate (PMS) has emerged as an exceptionally effective artificial electron shuttle in TBFCs. Yu et al. demonstrated that PMS could mediate high-efficiency charge extraction from thylakoids, yielding a photocurrent nearly 1000 times greater than that generated with para-benzoquinone (PBQ) [44]. The mechanism underlying PMS’s superior performance is rooted in its redox potential, which aligns favorably with ferredoxin and plastocyanin, the terminal electron carriers downstream of PSI. As illustrated in Figure 5c, upon illumination, PMS accepts electrons from these reduced species, undergoes rapid redox cycling, and delivers the electrons to the electrode. Importantly, PMS possesses high aqueous solubility, fast homogeneous electron transfer rates, and limited semiquinone formation, factors that collectively enhance charge separation efficiency and suppress recombination. However, photocurrent generation exhibited a concentration-dependent peak, after which performance declined due to diffusion limitations, indicating a need for careful mediator loading optimization.

In contrast to soluble mediators, immobilized redox-active compounds offer spatial confinement, proximity-driven electron transfer, and reduced diffusional losses. Lam et al. demonstrated the utility of a covalently anchored mediator system employing cystamine and pyrroloquinoline quinone (PQQ) on gold electrodes (Figure 5d) [45]. This self-assembled monolayer (SAM)-based strategy yielded a stable, electron-conductive platform for thylakoid membrane immobilization in micro-photosynthetic electrochemical cells (μPECs). The immobilized PQQ operates as a fixed-position redox relay that bridges thylakoid electron donors and the gold electrode, and the covalent immobilization restricts its movement, enabling rapid and localized electron transfer without reliance on diffusion. This architecture effectively mimics DET while maintaining the high reaction kinetics of MET. The SAM layer composed of cystamine ensures that the PQQ is properly oriented and electronically coupled, minimizing interfacial resistance and promoting efficient charge injection into the external circuit. The layer-by-layer immobilization was quantitatively verified using quartz crystal microbalance with dissipation, indicating the precision and reproducibility of the interface. This system demonstrated both enhanced photocurrent generation and improved structural stability, suggesting that such immobilized mediator platforms may offer a scalable pathway for TBFC development.

Redox mediator selection and surface modification strategies continue to be critical factors influencing the efficiency, stability, and scalability of TBFCs. Soluble mediators such as PMS and PBQ provide tunable redox potentials and high mobility, facilitating rapid electron extraction from deeply embedded sites within the thylakoid electron transport chain. Their performance hinges on precise control of redox alignment, concentration, and light exposure conditions. Conversely, immobilized mediators like PQQ offer structural robustness, enhanced device longevity, and pseudo-DET characteristics, especially when combined with nanostructured or chemically functionalized electrode surfaces. Importantly, the ability to design mediator systems through computational redox modeling, surface chemistry, and biointerface engineering has ushered in a new era of performance-optimized TBFCs. An emerging direction involves the integration of full PSI and PSII linear electron transport in biomimetic Z-schemes, enabling unassisted water splitting and directional charge flow.

## 4. Advancements in PSI- and PSII-Based Biofuel Cells and Biohybrid Architectures

Building upon the foundational role of redox mediators in facilitating electron transfer within TBFCs, a parallel and increasingly critical focus has emerged: leveraging the individual photosystems (PSI and PSII) as modular, tunable photobiocatalysts. While mediators provide kinetic assistance to overcome electron transfer bottlenecks, the intrinsic photophysical and electrochemical characteristics of PSI and PSII themselves are equally important in dictating device performance, durability, and application specificity. Recent innovations in PSI- and PSII-based architectures are pushing the boundaries of biohybrid energy systems by emulating the directional charge flow and catalytic precision observed in natural photosynthesis while overcoming constraints related to material compatibility, light capture efficiency, and system stability.

### 4.1. Photosystem I as a Bioanodic Catalyst

**Figure 6:**
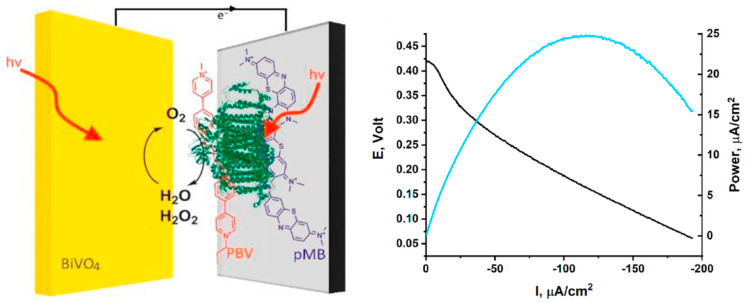
Schematic illustration and performance curves of PSI-based biofuel cells showing enhanced power output through PSI integration under illumination [46].

PSI is a robust, light-driven electron pump that operates at high quantum efficiency, capable of reducing ferredoxin and NADP^+^ in vivo. Structurally, PSI features a [4Fe-4S] cluster at its terminal acceptor site, enabling high-energy electron delivery upon photoactivation. In biofuel cell configurations, PSI’s long-lived charge-separated state and low overpotentials make it an attractive anodic catalyst. A central challenge, however, lies in achieving effective electron harvesting from PSI while maintaining its photochemical integrity outside the native thylakoid environment. To this end, integration strategies have focused on maximizing orientation control, surface immobilization density, and electrical connectivity. Efforts to improve PSI performance in bioelectrochemical systems have led to the development of hybrid photoelectrode architectures that marry natural photosystems with synthetic semiconductors. An example is the Z-scheme-inspired PSI photo-bioelectrochemical cell designed by Herzallh et al., which functions under ambient solar illumination without requiring external electron donors or applied bias (Figure 6) [46]. In this architecture, PSI was asymmetrically functionalized using polymethylene blue at the donor site and polybutyl viologen at the acceptor site to mimic the spatial separation of charge carriers found in natural photosynthetic Z-schemes. Coupled with a BiVO_4_/CoP photoanode, this configuration facilitated unidirectional charge flow and achieved a peak power density of 25 μW/cm^2^ exceeding the performance of conventional PSI/viologen-mediated systems.

Importantly, the design incorporated a dual-pathway strategy to mitigate oxidative stress caused by H_2_O_2_, a common degradation product when PSI operates in aerobic media. H_2_O_2_ was decomposed both photochemically at the BiVO_4_ photoanode and electrochemically at the polymethylene blue-modified PSI cathode. This multi-pronged stabilization strategy ensured sustained activity and extended device lifetime. Such semiconductor–photosystem hybrids illustrate the utility of co-engineering photophysical environments and redox landscapes to replicate the directionality, efficiency, and resilience of natural photosynthesis in bioelectronic systems.

### 4.2. Photosystem II in Bioelectrochemical Systems

PSII has gained traction in TBFC research due to its unique catalytic role in water oxidation and oxygen evolution. Functionally, PSII catalyzes the photolysis of water into electrons, protons, and molecular oxygen, driven by photon absorption at its chlorophyll-containing reaction center (P680). This makes PSII an ideal candidate for anodic integration in photo-bioelectrochemical cells aiming for self-sustained operation without sacrificial electron donors. However, unlike PSI, PSII is more susceptible to photoinhibition and oxidative degradation, necessitating precise environmental control and stabilization during biohybrid assembly. In practical applications, PSII has demonstrated significant potential in biosensing due to its sensitivity to environmental toxins, particularly herbicides that inhibit the QB site in the electron transport chain [47]. PSII-based sensors can detect picomolar levels of such contaminants by monitoring changes in chlorophyll fluorescence or photocurrent suppression. Beyond sensing, PSII has also been effectively integrated into photoanodes where it supports direct photoinduced oxygen evolution and current generation [48]. These devices eliminate the need for sacrificial donors, leveraging the water-splitting capability of PSII to sustain the redox cycle.

From a mechanistic standpoint, the water-oxidizing complex of PSII comprising a Mn_4_CaO_5_ cluster requires a delicate balance of hydration, pH, and light exposure to maintain its catalytic activity. Consequently, bioelectrode configurations incorporating PSII must optimize immobilization methods to preserve structural integrity while facilitating efficient charge transfer. Recent strategies include the use of protective polymer films, layer-by-layer deposition techniques, and orientation-specific attachment Via affinity tags or chemical linkers [49]. These methods aim to reduce photodamage, minimize recombination losses, and enhance overall turnover frequency.

Recent advancements have also leveraged structural and spectroscopic insights into PSII dynamics to fine-tune device performance. Fluorescence-based techniques such as variable chlorophyll fluorescence offer non-invasive tools for assessing PSII functionality, guiding the optimization of electrode design and environmental parameters [50]. Moreover, genetic engineering approaches that stabilize PSII assembly intermediates or confer resistance to photoinhibition are being explored to extend the operational lifetime of PSII-based bioelectrochemical devices.

The distinct photophysical and catalytic properties of PSI and PSII offer complementary advantages for next-generation TBFCs and biosensors. PSI’s high-efficiency electron injection and long-lived charge separation make it ideal for light-harvesting and charge relay in photoanodes or photocathodes, particularly when integrated with semiconductors for Z-scheme or tandem architectures. Conversely, PSII’s ability to directly extract electrons from water introduces autonomy and sustainability into the system, with additional benefits for environmental sensing applications. Future optimization of PSI- and PSII-based devices will depend on continued advances in protein engineering, surface chemistry, and materials integration. Hybrid platforms that spatially organize both photosystems, recreating the full linear electron flow pathway from water to NADPH are particularly promising. Such designs could enable artificial photosynthetic systems that not only emulate but enhance natural energy conversion processes, paving the way for solar-driven fuel generation, pollutant sensing, and biocompatible power sources in medical and environmental technologies. To further assist readers in evaluating the relative strengths and trade-offs across material classes, Table 1 summarizes representative TBFC configurations, highlighting key materials, architectures, and performance metrics reported in the literature.

The comparative analysis reveals that hybrid systems particularly those combining thylakoid membranes or PSII with engineered nanostructures like indium-tin-oxide nanoparticles, osmium redox polymers, or RuO_2_ tend to achieve the highest reported photocurrent densities (up to 500 µA/cm^2^) and power outputs (>100 µW/cm^2^) [20,24]. Full thylakoid-based TBFCs generally show moderate performance (50–100 µA/cm^2^), with improved stability when interfaced with 3D carbon materials like CNTs or rGO [4,17]. PSI-only systems exhibit efficient electron injection but lower overall output due to the absence of water oxidation, while PSII-only platforms offer oxygen evolution functionality but face challenges in electron transfer continuity. Systems using conductive polymers (e.g., PEDOT, PAni) yield moderate output (15–40 µA/cm^2^) but are attractive for their printability and mechanical flexibility. These trends indicate that combining targeted photosystem extraction with engineered nanomaterials currently offers the best pathway toward high-efficiency and scalable TBFCs, whereas unmodified membrane systems may be more appropriate for low-cost, disposable applications.

## 5. Outlook and Future Perspectives on TBFCS

TBFCs have emerged as an alternative platform for sustainable energy conversion, leveraging the intrinsic light-harvesting and electron transport mechanisms of photosynthetic systems. Advancements have been achieved through the integration of functional nanomaterials such as RuO_2_, CNTs, and MXenes, which enhance electrode conductivity, facilitate charge separation, and expand the effective surface area for bioelectrode interactions. In parallel, novel bioelectrode architectures featuring strategic surface modifications and 3D conductive scaffolds have enabled partial DET between thylakoid membranes and electrode surfaces, reducing reliance on soluble redox mediators and enhancing system biocompatibility. Hybrid platforms incorporating thylakoid membranes with photoactive semiconductors or enzymatic cathodes have also yielded promising gains in photocurrent density and energy conversion efficiency. However, despite this progress, TBFCs remain largely confined to proof-of-concept demonstrations, hindered by persistent biochemical, material, and systems-level challenges that limit their operational robustness, scalability, and long-term functionality.

### 5.1. Challenges in Stability and Biointegration

A primary barrier to the practical application of TBFCs is the limited operational stability of isolated thylakoid membranes. These protein–lipid complexes are highly vulnerable to photoinhibition, desiccation, oxidative stress, and thermal or pH fluctuations [51]. Light-driven generation of reactive oxygen species (ROS) exacerbates degradation, leading to rapid declines in photocurrent and membrane integrity [52]. Although protective immobilization matrices such as albumin–glutaraldehyde crosslinked films have shown partial success in resisting environmental perturbations, they fail to provide sustained protection under extended illumination or dynamic ambient conditions [51]. Additionally, the electron transfer efficiency remains suboptimal due to the complex multilayer architecture of thylakoid membranes. The spatial separation of redox-active sites from the electrode interface, combined with diffusional limitations of protons, ions, or endogenous mediators, hinders efficient DET. Although the use of high-surface-area nanostructures (e.g., micropillar arrays, inverse opals, or 3D-printed lattice electrodes) has improved the physical interface and enhanced electron harvesting [5,53], the mechanical fragility of bioelectrodes and their tendency toward delamination during operation remain unresolved. Further, the integration of soft biological assemblies with rigid synthetic materials introduces additional complications in terms of mechanical mismatch and loss of biofunctionality. These imperfect surface chemistries and inadequate immobilization strategies can compromise the native structure of photosystems, reduce light absorption efficiency and limit charge carrier lifetime [54]. Moreover, biofouling, nonspecific adsorption, and variable protein orientation further reduce reproducibility and performance.

Beyond biochemical fragility, TBFCs face significant challenges in scalable fabrication. The yield and purity of biologically derived components particularly thylakoid membranes and photosystem subcomplexes can vary significantly between preparations, leading to inconsistent device performance. Current device architectures often rely on batchwise assembly or custom fabrication techniques, which are poorly suited to large-scale or automated production. In addition, long-term storage stability remains a pressing concern, as isolated thylakoid membranes lose photoactivity rapidly in the absence of stabilizing media or continuous hydration.

### 5.2. Emerging Strategies and Opportunities

To advance TBFCs toward practical deployment, several critical areas require concentrated research and development. First, the encapsulation and stabilization of thylakoid membranes remain essential for preserving hydration and maintaining long-term photoactivity. Innovative encapsulation approaches such as hydrogel matrices, artificial vesicles, or vapor-deposited coatings must be developed to mitigate ROS-induced degradation and support ambient storage without compromising the functional integrity of light-harvesting complexes. Integration of ROS-scavenging enzymes, such as catalase or superoxide dismutase has demonstrated moderate success in reducing photodamage but requires further refinement to maintain enzyme stability and activity under device-relevant conditions [52]. Additionally, enhancing oxidative stress resistance through molecular engineering of photosynthetic complexes presents a promising avenue. This could involve redesigning proteins, incorporating synthetic analogs of essential cofactors, or implementing feedback mechanisms that dynamically regulate electron flow to reduce photodamage under high-light conditions. Such approaches would significantly improve operational lifetime and robustness across diverse environmental settings.

Genetic engineering of photosynthetic organisms or synthetic reconstruction of robust variants of the oxygen-evolving complex presents another opportunity to enhance photostability and broaden operational tolerance to environmental extremes [55]. Meanwhile, biohybrid electrode materials incorporating conductive polymers such as PEDOT:PSS or polyaniline offer synergistic advantages in charge mobility, flexibility, and interface compatibility [29]. A particularly exciting avenue is the use of living photosynthetic organisms such as cyanobacteria or microalgae as dynamic and self-repairing biofactories within TBFCs [56]. These systems offer inherent adaptability, extended lifespan, and the ability to regenerate damaged components, providing a potential route to self-sustaining and robust bioelectrochemical platforms.

Furthermore, the systematic exploration of redox mediator engineering, particularly the use of organic-inorganic hybrids, ionic liquids, or electroactive hydrogels, may unlock new avenues to control charge transport across biohybrid interfaces. Coupled with real-time monitoring tools, such as in situ electrochemical impedance spectroscopy or fluorescence microscopy, these advances will enable a deeper understanding of degradation pathways and inform the rational design of more resilient TBFC systems. Improved electrode–biointerface engineering is also paramount. Fabricating multiscale electrode architectures with tunable surface chemistry, optimized nanostructures, and biomolecular tethering strategies will be critical to achieving stable, high-efficiency DET without denaturing sensitive protein structures. These interfaces must support strong adhesion, reduce diffusional resistance, and maintain biological activity throughout prolonged operation. In terms of device scalability, standardizing thylakoid membrane extraction and purification protocols is vital for minimizing batch-to-batch variability. Integration of these biological components with scalable fabrication methods such as inkjet printing, microfluidic patterning, or roll-to-roll processing will help bridge the gap between laboratory prototypes and mass-producible devices.

Finally, future system integration efforts should focus on the development of modular, wireless, and potentially self-powered TBFC architectures. Such systems must be tailored for decentralized applications in biosensing, environmental monitoring, or remote power generation. Combining intelligent energy management strategies with miniaturized, multifunctional platforms will pave the way for TBFCs to serve as viable components in next generation bioelectronic technologies.

## 6. Conclusions

Over the past decade, substantial progress has been made in advancing TBFCs through a deeper understanding of the fundamental principles governing light-driven electron transfer in photosynthetic membranes. This knowledge has catalyzed innovations in device architecture, material integration, and electrochemical interfacing, enabling notable improvements in photocurrent generation, stability, and functional versatility. In particular, the incorporation of advanced electrode materials including carbon-based nanostructures, conductive polymers, and photoactive semiconductors has enhanced charge transfer kinetics, supporting both DET and MET pathways between thylakoid membranes and electrodes. The deliberate selection and orientation of photosynthetic components, such as the integration of PSII for oxygen evolution and PSI for efficient downstream electron harvesting, have further optimized redox coupling and improved overall energy conversion efficiencies. Hybrid systems that integrate thylakoids with semiconductor photoanodes, enzymatic or microbial cathodes, and printable electronics have expanded the operational scope of TBFCs. These configurations not only emulate key functional aspects of natural photosynthesis but also enable novel applications in artificial photosynthesis, CO_2_ fixation, hydrogen evolution, and biosensing. Importantly, these biohybrid devices are increasingly being developed on flexible substrates using low-cost and environmentally benign materials, reinforcing their potential for sustainable and decentralized energy solutions.

Despite these promising developments, TBFCs continue to face significant challenges, particularly related to the operational stability of biological components, the complexity of maintaining functional bioelectrode interfaces, and the scalability of device fabrication. Issues such as ROS-induced degradation, desiccation, and poor long-term performance under real-world conditions emphasize the need for more robust encapsulation techniques, bioengineering strategies, and system-level integration. Moreover, the variability inherent in biologically derived components presents a barrier to reproducibility, which must be addressed to enable the translation of TBFCs from lab-scale prototypes to commercial applications. Overcoming the current limitations through thoughtful design, scalable production, and rigorous systems-level evaluation will be essential to transitioning TBFCs from laboratory to impactful components of the future green energy landscape. Nonetheless, the progress reviewed here highlights the viability and versatility of TBFCs as a next generation platform for bioelectronic energy harvesting.

## Figures and Tables

**Table 1:** Summary of representative thylakoid-based biofuel cell (TBFC) configurations.

No.	Electrode Material	Architecture	Electron Transfer Mechanism	Photocurrent Density (µA/cm^2^)	Power Output (µW/cm^2^)	Reference
1	MWCNTs + Thylakoids	Molecular tethered 3D network	(DET)	68	5.3	[4]
2	CNTs with MAP coating	Percolation biofilm	(DET)	4.25	Not reported	[12]
3	Aminoaryl-functionalized rGO	3D structured matrix	(DET)	5.24 ± 0.5	1.79 ± 0.19	[18]
4	TiO_2_ + Thylakoids	Semiconductor photoanode	(MET) Viologen	~50	Not reported	[20]
5	RuO_2_ nanosheets	Layered planar electrode	(DET)	~100	~40	[22]
6	MnO_2_ on 3D-printed graphene	Porous photoanode scaffold	(DET)	580	93	[23]
7	Graphite + ITO NPs + Os polymer	Composite with optimized stoichiometry	(MET) Os-redox polymer	500	122	[24]
8	LIG + PEDOT	Multidimensional, flexible bioanode	(DET)	18	36	[28]

## Data Availability

No applicable.
